# 'Rumours' and clinical trials: a retrospective examination of a paediatric malnutrition study in Zambia, southern Africa

**DOI:** 10.1186/1471-2458-10-556

**Published:** 2010-09-17

**Authors:** Patricia Kingori, Maureen Muchimba, Bornwell Sikateyo, Beatrice Amadi, Paul Kelly

**Affiliations:** 1 Anthropologies of African Biosciences, Faculty of Public Health and Policy, London School of Hygiene and Tropical Medicine, Tavistock Place, London WC13 7HT, UK; 2 School of Public Health, Department of Epidemiology, University of Alabama,1665 University Blvd, Birmingham, AL 35294, USA; 3 Tropical Gastroenterology and Nutrition Group, University of Zambia School of Medicine, University Teaching Hospital, Lusaka, Zambia; 4 Barts & The London School of Medicine, Queen Mary University of London, Turner Street, London E1 2AD, UK

## Abstract

**Background:**

Many public health researchers conducting studies in resource-constrained settings have experienced negative 'rumours' about their work; in some cases they have been reported to create serious challenges and derail studies. However, what may appear superficially as 'gossip' or 'rumours' can also be regarded and understood as metaphors which represent local concerns. For researchers unaccustomed to having concerns expressed from participants in this manner, possible reactions can be to be unduly perturbed or conversely dismissive.

This paper represents a retrospective examination of a malnutrition study conducted by an international team of researchers in Zambia, Southern Africa. The fears of mothers whose children were involved in the study and some of the concerns which were expressed as rumours are also presented. This paper argues that there is an underlying logic to these anxieties and to dismiss them simply as 'rumours' or 'gossip' would be to overlook the historic and socio-economic factors which have contributed to their production.

**Methods:**

Qualitative interviews were conducted with the mothers whose children were involved in the study and with the research nurses. Twenty five face-to-face interviews and 2 focus group discussions (FGDs) were conducted with mothers. In addition, face-to-face interviews were conducted with research nurses participating in the trial.

**Results:**

A prominent anxiety expressed as rumours by the mothers whose children were involved in the study was that recruitment into the trial was an indicator that the child was HIV-infected. Other anxieties included that the trial was a disguise for witchcraft or Satanism and that the children's body parts would be removed and sold. In addition, the liquid, milk-based food given to the children to improve their nutrition was suspected of being insufficiently nutritious, thus worsening their condition.

The form which these anxieties took, such as rumours related to the stealing of body parts and other anxieties about a stigmatised condition, provide an insight into the historical, socio-economic and cultural influences in such settings.

**Conclusions:**

Employing strategies to understand local concerns should accompany research aims to achieve optimal success. The concerns raised by the participants we interviewed are not unique to this study. They are produced in countries where the historic, socio-economic and cultural settings communicate anxieties in this format. By examining this study we have shown that by contextualizing these 'rumours', the concerns they express can be constructively addressed and in turn result in the successful conduct of research aims.

## Background

For many potential participants of medical research in developing countries, being asked to enrol in a clinical trial can present a dilemma[1]. On the one hand, medical research may offer access to enhanced medical and nursing care, whilst on the other, it may be the subject of suspicion and fear. Previous studies suggest that these anxieties may become manifested as 'rumours' during the conduct of research[2,3]. These 'rumours' can disrupt the recruitment and retention of study participants and have in some cases derailed studies altogether [4-6].

In this article, we discuss the fears of mothers whose children were involved in a malnutrition study conducted by an international team of researchers in Zambia, Southern Africa. In doing so, this paper presents a case study of how deep-seated suspicions can challenge medical research and public health interventions. However, it also argues that there is an underlying logic to these anxieties and to dismiss them simply as 'rumours' or 'gossip', would be to overlook their significance.

This paper represents a retrospective examination of the conditions in which the trial took place. This includes some of its the practical and logistical features, together with the historic and socio-economic contexts of the study, all of which have led to a setting which was fertile to concerns and 'rumours'. In addition, such observations allow for other researchers conducting studies in similar conditions to undertake a pre-emptive approach to reduce the challenges which such concerns, expressed as 'rumours', can create.

### The Study Setting

In Zambia, poor nutrition is significantly associated with childhood morbidity and mortality, and reduced adult life expectancy and economic performance [7]. In 1999 (a year into the study we present), the under-five mortality was estimated to be 187 per 1000 live births, compared to 159 in the Sub-Saharan region. In the same year, life expectancy at birth was 38.5 years compared to 46.8 in the region[8]. In addition to inadequate nutrition, HIV/AIDS, tuberculosis (TB) and malaria have also been leading causes of morbidity and mortality in the past decade, with diarrhoeal disease and malnutrition being common in children with AIDS[9]. The trial which was carried out was designed to determine whether liquid, milk-based foods containing small peptides and simple sugars would allow easier absorption and assimilation and cause fewer hypersensitivity problems in severely malnourished children.

#### The PDM study design and conduct

Children were included if they had persistent diarrhoea (diarrhoea lasting 14 days or more) with malnutrition. Mothers of children presenting with persistent diarrhoea and malnutrition (PDM), were then approached by the research nurses who explained the study protocol and sought their consent to have their children participate in the trial. If consent was given, the mother was interviewed, using a standard questionnaire and the children were examined by both nurses and doctors. The hospital treatment was initiated with rehydration, antibiotics, vitamins and minerals in a fairly intensive environment. In addition, the children were randomised either to a standard nutritional rehabilitation programme (skimmed milk-based food) or to exclusive use of a complete infant elemental milk-based food (Neocate, SHS Ltd, Liverpool, UK). Initially, both diets were liquid, but the children allocated to the standard nutritional rehabilitation group were gradually switched to solid soy/maize porridge in the second week of treatment [9]. The randomisation of the children into the different study arms was undertaken by the doctors and nurses involved in the trial, who opened sealed envelopes according to a strictly sequential numbering of each child recruited.

One hundred children were given Neocate and 100 given the diet based on skimmed milk with the soya/maize blend porridge (routine group). All of the children were given vitamin and mineral supplements and other standard treatments. Once the trial started, children also underwent endoscopy with duodenal biopsies. This took place under sedation to assess intestinal damage, search for parasitic infection, and detect for allergies. Further details of the trial have been reported elsewhere [10].

#### PDM study

The PDM study took place on the malnutrition ward, A07, in the University Teaching Hospital (UTH), Lusaka, Zambia. The ward is divided into four bays. Bay 1 is the reception bay, also known as the "acute bay", which is where malnourished children are placed shortly after admission. Depending on progress made, the children are moved from the first through the fourth bay, before they are discharged. The children are normally treated in the ward over a period of two to four weeks. A description of the conditions on the ward was described by the one of the nurses in a UNICEF article entitled *The Struggle to Survive in Ward AO7*[11], as follows;

"There are 59 beds, but sometimes we have 90 to 100 patients. They have to share cribs in Bay 4, the rehabilitation bay where the children go before they are discharged. We can't send them away, so the children must share resources..."

## Methods

In order to understand the mothers' perceptions and reactions to the trial, face-to-face in-depth interviews and focus group discussions (FGDs) were held during the course of the study. In keeping with qualitative research techniques, the sampling for the interviews was undertaken purposively and was framed while researching the source of the rumours among the mothers. As a consequence, the numbers of interviews conducted were smaller than those involved in the main PDM study but were sufficient to provide an insight as to concerns of mothers.

Twelve face-to-face interviews were conducted with mothers of children recruited into the trial and 13 with mothers of children ineligible for inclusion in the trial. Two focus groups were also undertaken. Five of the 10 nurses who were participating in the trial were also interviewed at the same time; they had been asked to record their observations as the study progressed, and these records were analysed. The interviews were transcribed and analysed. These interviews were approved by the research ethics committee of the University of Zambia School of Medicine.

## Results

### The 'Rumours'

During the course of the trial it was observed that mothers who were approached about the trial had some concerns about consenting to the inclusion of their children. Almost 20%, of the mothers who were approached declined to participate and cited one of the rumours as their reason for not giving their consent[10]. This often necessitated several discussions with each mother to explain the concepts of nutritional rehabilitation and the need for the study. Initially, some mothers consented (approximately 10%), only to withdraw after discussing the study with the mothers whose children did not meet the inclusion criteria. Other mothers agreed to participate but were later found to be non-compliant with the intervention (e.g. by throwing away food).

One common reason cited for withdrawal was the fear that their children would be given a novel milk-based feed. Another reason advanced was the fear of Satanism, based on information that the blood specimens collected as part of patient evaluation were being sold to Satanists and would result in death of their children. As the trial progressed, the intensity and content of some of these concerns grew, requiring the research team to give them greater attention than initially anticipated.

In relation to this clinical trial, the main concerns expressed through rumours were that:

1. Recruitment was really an indicator that the medical and nursing staff knew that the child was HIV-infected

2. The trial was a disguise for witchcraft or Satanism

3. The children's blood and body parts would be removed and sold, presumably for use in witchcraft

4. The liquid, milk-based food given as part of the study would worsen the condition of the child because it was believed that a child simply could not survive on liquid food only

The possible cause and justification for these concerns and the impact that they had on the trial will be examined in turn.

### Recruitment was really an indicator that the child was HIV-infected

During the FGDs with nurses, it was reported that some of the mothers raised their suspicions that the blood taken from their children was being secretly tested for HIV. They felt that this was the criterion on which treatment allocation was decided. Some mothers formed the belief that the elemental milk-based food was used as an immune booster for HIV-infected children. This belief interacted with the stigma of HIV and led to some mothers requesting that their child be switched to the other treatment arm to avoid being associated with the HIV illness.

### The PDM study staff were involved in witchcraft or Satanism

Both blood sampling and the taking of endoscopic biopsies for histology were viewed suspiciously. The nurses and other staff were aware of concerns that such samples were used by the staff for purposes of witchcraft, and that blood taken from the children could be sold or, worse, used in witchcraft. Some mothers suggested that the doctors and nurses on the study were Satanists. Another concern was that even the smallest volumes of blood taken from the children during the study would enfeeble them.

### The children's body parts would be removed and sold

The endoscopic procedures were a significant source of anxiety. Some of the mothers were concerned that during the first endoscopy, small pieces of the child's intestines were removed. These would then be used in medical students' lessons or sold to drug manufacturing companies. The purpose of the second endoscopy was undertaken by doctors to ensure that the intestines cut during the first procedure had healed. If unsuccessful, then other endoscopies would follow. It was feared that it was these endoscopic procedures which necessitated the month-long stay in the ward.

### The liquid, milk-based food being given would worsen the condition of the child

Initially, the mothers were sceptical about both the Neocate and skimmed milk diets because they believed they were insufficient. Some mothers felt that their children could not survive solely on the milk diets and the nurses recounted many instances of mothers secretly giving them other foods brought from home. One child, who later died, was given freshly squeezed orange juice by his mother. She told the other mothers that the child needed vitamins since he would die of hunger if fed on "milk" only. However, when it was observed that the children being given Neocate made good progress, some of the other mothers asked for Neocate as well.

Another concern related to the milk diets was that it would worsen the diarrhoea, so in the early days of the trial some mothers were discovered to have been discarding it. Some mothers disliked the smell and taste of Neocate and were found to have thrown it away for this reason. It was observed by the study team that Neocate, (which has a bitter taste to most adults) was taken readily by the children. Other mothers were willing to accept the necessity of the liquid-only diet after explanation from the nurses, but influence from relatives during visiting hours sometimes dispelled any assurances they might have had.

Furthermore, oxygen therapy became very controversial. Mothers soon realised that children receiving oxygen had a high mortality rate, as these were the most seriously ill children, and the association led them to refuse oxygen for their children as they believed it must be toxic.

## Discussion

Rumours as to the purpose and outcome of research is a common feature of medical research conducted in developing countries [12]. For instance, common rumours are of 'blood or organ stealing', which have been reported in Africa, South America, Russia and parts of Eastern Europe [13,14]. For Geissler and Pool, rumours are best appreciated as commentaries on social relations, involving and extending beyond scientific medical research [2]. As such they are best understood as metaphors, representing local concerns and should be considered within this context [15].

### The historical context to the fears surrounding this trial

Suspicions about western medicine and health interventions in Zambia have existed long before this trial commenced. There is a long and well-documented history of rumours related to medical intervention, research and 'blood stealing' in Zambia [3]. Musambachime suggests that rumours are most likely to exist in areas where there is already a history of communicating concerns through this medium. He argues that Zambia is "*fertile ground for these rumours*" (p.203) based on distrust generated by poorly administered colonial interventions [16]. White argues that in addition to the vampire men (Banyama), who were accused of stealing blood, there were other rumours related to food and its supply in Zambia between 1930-1964[17].

Musambachime also notes the continuation of rumours as a medium of concerns and that in post-colonial times strangers could potentially be the subject of the accusations if their behaviour were viewed to be unfamiliar.

"*European doctors, fat administrators, prospectors, surveyors, and tourists were highly suspected of being Banyama. Rumours were also fuelled by appeals for and witnessing of blood donations or transfusions or seeing pictures of either, and by stories of mysterious disappearances of people in a given area. These rumours, which though unverified were believed and spread by many in a distorted form, bred fear and insecurity*." (p.208)

For Lungu, these rumours were often fuelled by a desire for meaning, a quest for clarification, or the search for a logical explanation of an event, which were witnessed to be more prevalent in times of crisis. The value of these historical accounts to the experience of the PDM trial is that they are able to provide greater insights into the production of rumours and the conditions which foster them. Musambachime also suggests rumours are heightened by socio-economic and political factors [17-19].

### The socio-economic context

The political environment of Zambia has been stable for the past four decades, especially in comparison to some of its neighbours, such as Zimbabwe. However, it is estimated that more than 80% of the population existed below the poverty line at the time of the PDM study [20-24]. Most of the mothers who participated in the PDM study were involved in informal self-employment, trading in consumables or other semi-skilled trades[20]. Having children who were hospitalised meant that some of these mothers could not participate in contributing towards their family income. This generated great pressure for the women and children to leave the hospital at the earliest possible opportunity. The financial implications in addition to emotional stress of a sick child were important factors in this study.

### Witchcraft accusations

The nurses interviewed reported that many of the mothers preferred explanations of witchcraft to the biomedical accounts that they presented. Traditional beliefs are widely held about the causes and cures of illnesses, including belief in traditional medicine and witchcraft [21]. Witchcraft is often perceived as the cause for sickness and death, especially in cases of HIV/AIDS and a key feature in the witchcraft is the possession of body parts or blood [22]. Sporadically, there are accusations of theft of organs and blood for the purposes of witchcraft. For instance, in March 1995, three years before the study began, rioting erupted in Mazabuka after a businessman was accused of offering high rewards for children's organs for witchcraft, 'money making rituals' and business enhancing rites[21]. Six months later alleged trade in human organs sparked another riot and racial attacks on Zambians of Asian descent in Livingstone [22].

Comaroff and Comaroff argue that these rumours were expressions of concern with the exploitation and appropriation of bodies by people with power and/or knowledge [22]. They argue that they are concerned with processes of globalization and the forms of exploitation associated with it. However, accusations of witchcraft are also seen as attempts to push the blame and the stigma of certain conditions away from the individual(s). Some mothers felt that having a child who had malnutrition would cast doubt on how they cared for their child.

### Stigmatised conditions

In this study, there appeared to be stigma associated with having a malnourished child and also with being labelled as having HIV/AIDS. Many mothers denied that their children were malnourished, even though objective measures employed provided evidence to the contrary. Some mothers did admit that they had difficulty feeding their children as they would like due to economic or social circumstances. As an example of this, one mother said she had to be at the market all day selling, and this left her insufficient time to make sure her child was properly fed. In discussions of the cause of the children's problems, there appeared to be differences between mothers of children recruited or not recruited. The former appeared to be more willing to accept the role of "hunger", "malnutrition", or "kwashi" (kwashiorkor). The latter group preferred to believe that the illnesses were due to too much dust in the townships or too much rain in the past rainy season. These mothers appeared to be offended by the insinuation that their children were ill due to "hunger" and some implied that the nurses attached this pejorative label without examining the children properly.

During the conduct of the trial, the HIV prevalence in Lusaka was approximately 22%[7]. Therefore, the concerns by the mothers about HIV/AIDS and blood tests for the study were within this context. Bond *et al*. argue that the old stigmas associated with TB, diarrhoea and skin rashes were either accentuated or layered upon new stigmas because of HIV [23-25]. So the stigmatised conditions of diarrhoea and malnutrition suffered by the children and their subsequent hospitalisation were layered upon the stigma of having HIV.

### The subject of the trial

Paediatric medical interventions can be emotive in both the developed and the developing world [26,27]. However, paediatric medical research in developing countries can present a variety of ethical and cultural challenges [1]. This study involved a nutritional intervention and the subject of food and feeding were sensitive issues for the families and communities involved. For instance, in a paediatric trial in Malawi, drinks and biscuits were given to mothers while they were waiting for their children to be weighed. This food came to symbolise the mothers concerns about the trial and was rumoured to be an attempt to poison infants through their mother's milk [1].

Concerns about biomedical interventions conducted on children can also be based on the legacy of poorly conducted medical research and interventions in the region. For instance, in 1995, three years before this study began, Richard McGown, a Scottish anaesthesiologist, was accused of five murders and convicted in the deaths of two infant patients whom he injected with lethal doses of morphine in Zimbabwe [28]. Whilst it is unlikely that such events directly influenced the behaviour of the mothers in this study, they appear to support some of the suspicions of medical research as legitimate and their legacy might influence the decision of mothers asked to participate in future studies.

### Health-seeking behaviour associated with PDM trial

The mothers involved in the trial appeared to adopt a pattern of health-seeking behaviour involving three stages. Initially, traditional medicines and remedies (i.e. non-biomedical interventions) such as guava leaves were used by the mothers or their relatives to treat the sick child. If this was unsuccessful then the mothers and/or their relatives sought medical care at the local clinic or a private health centre. If the symptoms persisted, the mothers were then referred to UTH. Health-seeking behaviour tends to follow this general pattern because traditional medicines and local health care facilities were often cheaper and more easily accessed [29].

However, this pattern had two main consequences for the treatment of patients and in turn for the conduct of the trial. Firstly, it meant that the cases of malnutrition with persistent diarrhoea were very severe by the time the children were admitted to UTH. As a consequence, invasive procedures were sometimes necessary, for example, the use of nasogastric feeding. The severity of the cases referred to the hospital meant that the mortality rate on the ward was high. Thirty-nine of the 200 children recruited during the course of this study died (19.5% case fatality rate). Secondly, it also meant that the anxiety levels in the mothers were typically raised and many mothers feared the outcome of interventions carried out on the ward (including oxygen). The high mortality rates contributed to the general feelings of apprehension.

### The conditions on the malnutrition ward

The paediatric wards at UTH during the conduct of this study were often congested. 'Floor beds', which were temporary beds, could be found on the wards due to the large number of patients and insufficient resources. In the malnutrition wards, patients usually shared 2-3 per cot bed or 4-5 on bigger hospital beds. Further to this, was the impact on the mothers caused by physically living on the ward while their children were hospitalised. In the malnutrition ward, high mortality rates, cramped conditions and the mothers in an emotionally-charged state created considerable scope for the generation and dissemination of 'rumours'.

### How can we best understand and manage these concerns?

The description of the context in which the PDM trial was conducted represents the conditions very often found in developing countries. The interaction of historical, socio-economic and clinical conditions, together with local belief systems and the fact that this study involved children can create circumstances which generate concerns. However, the anxieties discussed in this paper are not unique to Zambia and they have shaped the conduct and outcome of a number of studies. As such, examining the factors which give rise to their occurrence may present researchers with the opportunity to anticipate and manage their influence in future studies.

If, as Lungu suggests, these 'rumours' were a quest for information, then they place additional responsibilities on the part of researchers. This might mean providing explanations about the research and its procedures several times throughout the course of the trial. In addition, it also means approaching 'informed consent' as a continuous process, rather than a single event at the start of a trial. It also means providing information in a format shaped by the context of the study and this might involve including significant family members, not directly involved in the study. In this instance, it was the children's grandmothers who were influential but they were not included in the consenting process.

It is also important to pay attention to the relationship between the participants and the mediators of the research. In this study, the mediators were the nurses but it is often fieldworkers and research assistants. Their role in the successful running of trials and in dispelling concerns has been reported elsewhere and with adequate resources could make a difference in reducing the severity and the duration of the fears (Davis et al, 2004).

In addition, strategies which allow for study staff to be prepared for these types of concerns with training to manage them would be valuable. Many experienced field level staff may have acquired this knowledge through their involvement in numerous studies. It would strengthen studies if these experiences were provided with an outlet which could be incorporated into the study design. Such inclusions into the design of studies would involve re-examining many existing relationships with both study staff and research participants.

### Understanding the function of 'rumours'

According to Washington (2007), writing in the New York Times:

".... By continuing to dismiss their [African patients] reasonable fears, we raise the risk of even more needless illness and death."

**Figure 1:**
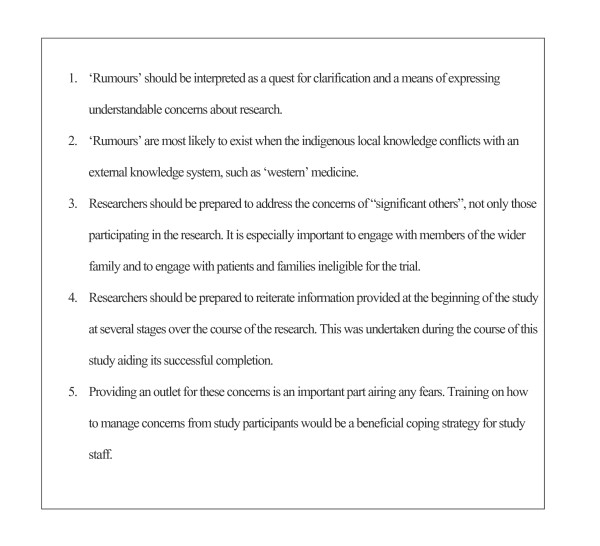
Lessons learned and recommendations

Geissler and Pool add that rumours should not be interpreted as ignorance or a lack of knowledge but rather as a means of expressing understandable concerns [2]. However, getting to the heart of the anxieties can present difficulties. Researchers unaccustomed to having concerns expressed from participants in this manner can be unduly perturbed by paying too much attention to the specifics of the medium of 'rumours'. In contrast, researchers can be dismissive of rumours, without realising the damage that ignoring them can cause to the conduct of their research. The PDM study has shown that by addressing the mothers' concerns the trial was completed as planned. Furthermore, by involving social scientists and employing the use of methodologies such as focus groups discussions, can provide an outlet for these anxieties and in turn allow them to be addressed appropriately (Figure 1).

## Conclusion

Reducing the levels of malnutrition among children and adults continues to be a challenge in Zambia and other parts of the developing world. As such, employing strategies to understanding local concerns must go hand-in hand with interventions in order to achieve optimal success. The concerns raised by the participants we interviewed were not unique to this study. They are produced in countries where the historic, socio-economic and cultural settings communicate anxieties in this format. By examining this study, we have shown that by contextualizing these 'rumours', the concerns they express can be constructively addressed and in turn result in the successful completion of interventions.

## Abbreviations

FGD: Focus Group Discussion; RCT: Randomised Control Trial; UTH: University Teaching Hospital; PDM: Persistent Diarrhoea and Malnutrition.

## Competing interests

The authors declare that they have no competing interests.

## Authors' contributions

PK and BA designed the study and drafted the manuscript. MM participated in data collection and analyses along with PK. PK, MM, BS, BA and PK drafted the manuscript. All authors read and approved the final manuscript.

## Pre-publication history

The pre-publication history for this paper can be accessed here:

http://www.biomedcentral.com/1471-2458/10/556/prepub
